# Characteristics of Side Effects in Non-Emergency Indications Using Computer-Controlled Pressurized Monoplace Hyperbaric Chambers: A Retrospective Multicenter Study

**DOI:** 10.3390/jcm13226835

**Published:** 2024-11-13

**Authors:** Hee-Young Lee, Soo Kang, Jin-Hui Paik, Tae-Kyu Ahn, Young-Ho Seo, Hyun Kim, Yong-Sung Cha, Yoonsuk Lee

**Affiliations:** 1 Department of Emergency Medicine, Yonsei University Wonju College of Medicine, Wonju 26426, Gangwon State, Republic of Korea; hylee3971@yonsei.ac.kr (H.-Y.L.); khyun@yonsei.ac.kr (H.K.); emyscha@yonsei.ac.kr (Y.-S.C.); 2 Department of Emergency Medicine, Inha University College of Medicine, Incheon 22332, Republic of Korea; drpeace@inha.ac.kr (S.K.); riven2ne@inha.ac.kr (J.-H.P.); jedwin@naver.com (T.-K.A.); louis7em@gmail.com (Y.-H.S.); 3 Research Institute of Hyperbaric Medicine and Science, Yonsei University Wonju College of Medicine, Wonju 26426, Gangwon State, Republic of Korea

**Keywords:** hyperbaric oxygen therapy, side effect, monoplace hyperbaric chamber

## Abstract

**Background:** Hyperbaric oxygen therapy (HBOT) involves inhaling nearly 100% oxygen in a pressurized environment and is commonly used to treat various diseases and injuries. Despite its well-known safety, HBOT is associated with side effects, with frequent middle ear barotrauma (MEB) and oxygen toxicity. Understanding the characteristics and risk factors associated with these side effects is critical for improving patient compliance and treatment outcomes. **Methods:** This retrospective multicenter study aimed to analyze the characteristics and factors associated with side effects during HBOT using a computer-controlled pressurized monoplace hyperbaric chamber. We conducted a retrospective observational study across the two tertiary hospitals in Korea, involving patients who received HBOT from October 2016 for one hospital and October 2017 for another hospital to June 2020. Data were extracted from electronic medical records and hyperbaric chamber logs, including patient demographics, medical history, HBOT indications, and details of side effects. Statistical analyses, including chi-square and *t*-tests, were used to compare variables. **Results:** A total of 247 patients (mean age: 59.35 ± 15.05 years, 63.56% male) were included. The most common indications for HBOT were sudden sensorineural hearing loss (27.94%) and post-graft/flap (24.29%). Hypertension (46.15%) and diabetes mellitus (39.27%) were the most frequent comorbidities. Otalgia was the most prevalent side effect (33.20%), followed by chest discomfort (2.02%) and headache (1.62%). A significant proportion of patients (11.74%) terminated HBOT due to side effects, with most pauses occurring at pressures between 1.2 and 1.4 ATA (26.67%). Side effects, particularly otalgia, significantly impact patient compliance with HBOT. **Conclusions:** The incidence of side effects varies by pressure level during treatment, suggesting the need for tailored strategies to minimize side effects. This study highlights the importance of patient monitoring and education to improve the safety and efficacy of HBOT in monoplace chambers.

## 1. Introduction

Hyperbaric oxygen therapy (HBOT) requires inhaling near 100% oxygen in an elevated pressure environment above 1.4 atmospheres absolute (ATA) [1]. Most studies report that the effect of HBOT requires pressure between 2.0 ATA and 3.0 ATA to obtain the clinical benefit [2]. There are two kinds of hyperbaric chambers. A monoplace chamber treats one patient per session, and a multiplace chamber can treat two or more patients together [3]. In Korea, HBOT is applied to the indications that are approved by the Korean National Health Insurance Service, which are the following: acute carbon monoxide poisoning, gas embolism, central retinal artery occlusion, decompression sickness, acute cyanide poisoning, tissue necrosis after radiation therapy, diabetic foot ulcer, after flap or graft, after amputation surgery, sudden sensorineural hearing loss, peripheral artery occlusion diseases (including Buerger’s disease), thermal burn, refractory osteomyelitis, intracranial abscess, gas gangrene, and hemorrhagic anemia [4]. Emergent diseases, such as acute carbon monoxide poisoning, arterial gas embolism, and decompression sickness, need prompt HBOT and only screen for the absolute contraindications of HBOT [5]. For non-emergent diseases, such as diabetic foot ulcers, ischemic wounds, chronic osteomyelitis, and sudden sensorineural hearing loss (SSNHL), HBOT is recommended after thorough assessments and adequate patient education to prevent the side effects [6]. HBOT is known to be safe compared to other therapies, and the incidence of side effects is low [7]. Barotrauma and oxygen toxicity are possible side effects of HBOT. During the treatment, barotrauma may occur when the compression starts to meet the therapeutic pressure [8].

Middle ear barotrauma (MEB) is the most common side effect of HBOT [9]. When pressure increases in the hyperbaric chamber, the tympanic membrane may be injured from the pressure difference. To prevent tympanic membrane injury, the Eustachian tube must be opened to equalize the inner and outer ear pressure [10]. According to previous studies, the incidence of MEB ranges from 8.9% to 65%, which depends on the skills of the hyperbaric chamber operator, the patient’s underlying conditions, and adequate patient education [11]. MEB may cause otalgic pain, decrease or loss of hearing, and rupture of the tympanic membrane [12]. The severity can be graded using the modified TEED scale and ranges from symptoms with no ontological signs (Grade 0) to a rupture of the tympanic membrane (Grade 5). If the injury is not severe, the tympanic membrane heals quickly with the pause of HBOT. The tympanic membrane may require weeks or months to recover, and permanent hearing loss may occur in severe cases. The incidence of sinus and pulmonary barotrauma is rare [13].

Compared to MEB, oxygen toxicity due to HBOT does not occur often. The most critical oxygen toxicity-related side effect involves the central nervous system (CNS) and a seizure. Oxygen toxicity may also involve side effects on the eye and lungs, which recover by stopping oxygen breathing in most cases [14]. Dr. Hadanny and his colleagues retrospectively reviewed 2334 patients who received HBOT from June 2010 to December 2014. Only one patient experienced oxygen toxicity-related seizures. Eight patients experienced myopia. The study only involved the patients who were treated in the multiplace chamber. In the multiplace chamber, two or more patients are treated together. Therefore, the rate and time required to increase the pressure in the chamber to the therapeutic range cannot be determined for individual patients [15].

The side effects of HBOT may determine the patient’s compliance with the completion of the treatment [16]. The treatment protocol for HBOT may vary by institution and nation. However, the protocol requires a minimum of 90 min of staying in the hyperbaric chamber for repetitive treatment sessions, depending on the indication [17]. Thus, establishing a strategy to prevent side effects is essential. Therefore, we have analyzed the time and pressure when the automated monoplace hyperbaric chamber paused or terminated the treatment due to the patient’s complaint about the side effects. By understanding the relationship between the side effects and a certain pressure, we could find the optimal compressing speed and be more cautious to prevent side effects. To prevent the side effects and increase the patient’s compliance with HBOT, we must understand the factors that cause the side effects in depth. This study analyzed the characteristics and aspects related to the side effects of HBOT in a computer-controlled pressurized monoplace chamber.

## 2. Materials and Methods

### 2.1. Study Design

**Figure 1:**
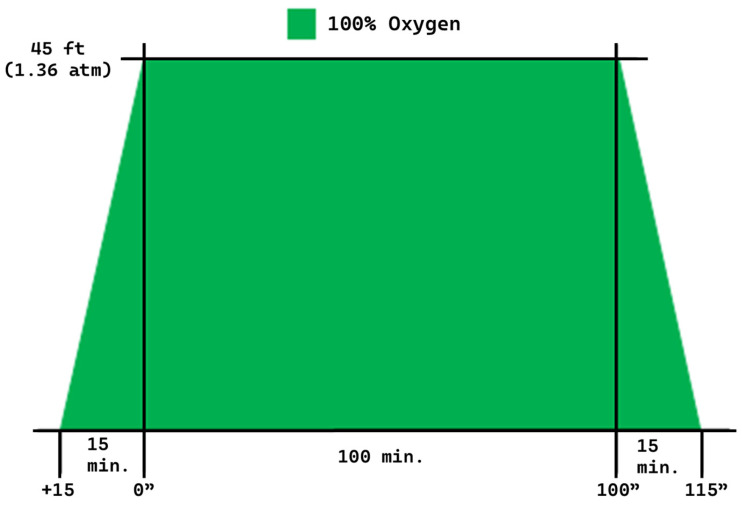
Clinical protocol used for non-emergent indications for both institutions.

This retrospective multicenter observational study includes patients from two tertiary-care academic hospitals, including hyperbaric facilities, Wonju Severance Christian Hospital (Wonju, Republic of Korea) and Inha University Hospital (Incheon, Republic of Korea). We retrieved the electronic data of how each hyperbaric treatment proceeded in the computer-controlled pressurized monoplace chamber (IBEX M2, IBEX Medical Systems, Seoul, Republic of Korea). The data included a real-time recording of treated and paused time and pressure changes. We matched the electronic records from the chamber and the electronic medical records with the patient’s hospital identification number. The Institutional Review Board of Wonju Severance Christian Hospital approved our study protocol (approval number: CR320074; approval date: 7 July 2020) and registered with Korea’s Clinical Research Information Service (CRIS: KCT0005974). The informed consent was waived for this study because it is a retrospective and observational study, and we conducted the study procedures under the Helsinki Declaration. The patient records and information were anonymized before analysis. Both institutions used the same clinical HBO protocols from the beginning (Figure 1). The protocol is slightly modified from reference with a descending time of 15 min [18].

### 2.2. Data Collection and Patient Characteristics

The nurse or paramedic who first instructed the patient to equalize their ear during decompression determined the patient’s alert and verbal status. They also provided the patients’ mental status to show that they could understand the instructions of the method to prevent barotrauma during the compression time.

The electronic medical records (EMR) included the following general characteristics: age, sex, HBOT indication; past medical history, including hypertension (HTN), diabetes mellitus (DM), and otologic; pulmonary, including asthma, chronic obstructive pulmonary disease (COPD), and pneumothorax; cardiovascular, renal, ophthalmic, cerebrovascular, psychological, and any malignant diseases; mental status at the initial treatment; and the outcome of the completed HBOT session. Past medical history has been categorized based on the ICD-10 code [19]. From the EMR, we also collected the patient’s clinical characteristics associated with HBOT, including a symptom that caused the pause of the treatment, HBOT session outcome at the initial trial, pressure at which the HBOT session paused with time-paused in minutes, and pressure at which the HBOT session terminated.

### 2.3. Statistical Analysis

From the collected data, we described the clinical characteristics of all study subjects and the number of HBOT pauses and treatment termination per hyperbaric treatment pressure grade. Data were collected and described as the number of events rather than the number of patients because treatment terminations occur independently once per patient, but an individual patient may have pauses in multiple pressure grades.

We selected specific statistical methods depending on the properties of the variables. Mean and standard deviation, or median and range, are used for continuous variables. Frequency and percentage are used for categorical variables. A chi-square test was used to compare 2 categorical variables, while categorical variables consisting of 3 or more categories were analyzed using Fisher’s exact test with the permutation resampling method for multiple testing adjustment. Two-sample *t*-tests or Mann–Whitney U tests were used to compare continuous variables. Normal distribution was analyzed using Student’s *t*-test. A *p*-value of <0.05 was considered statistically significant. We used SAS (version 9.4; SAS Institute, Inc., Cary, NC, USA) for all statistical analyses.

## 3. Results

### 3.1. Characteristics of Study Subjects

**Figure 2:**
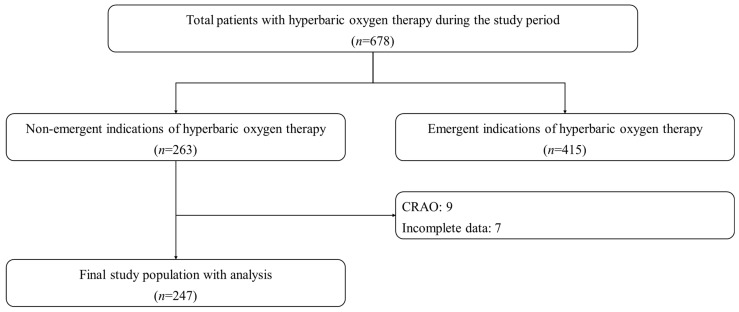
Flowchart for the total study population.

This multicenter retrospective registry-based observation study involved two tertiary university academic hospitals. Yonsei University Wonju Severance Christian Hospital (Wonju, Gangwon, Republic of Korea) included patients who received hyperbaric oxygen therapy (HBOT) from October 2016 to June 2020. Inha University Hospital (Incheon, Republic of Korea) included patients who received HBOT from October 2017 to June 2020. The total number of patients treated with HBOT in the monoplace chamber was 678, including 263 non-emergent indication patients. Among 263 cases, we excluded nine central retinal artery occlusion cases, with a specific treatment protocol requiring the pause during the compression time for vision check, and seven patients with incomplete data (Figure 2). To include more cases to analyze, we have included patients from when the automated monoplace hyperbaric chamber was introduced to hospitals.

The general characteristics of the subjects were analyzed. The average age was 59.35 ± 15.05. One hundred fifty-seven patients (63.56%) were male. SSNHL had sixty-nine cases (27.94%), the most frequent. Post-graft/flap was sixty patients (24.29%), and post-radiation therapy-induced tissue necrosis was thirty-one (12.55%), second and third in the order. Hypertension (114, 46.15%) was the most frequent in past medical history, and diabetes mellitus (97, 39.27%) was the second. One hundred thirty-seven patients (55.47%) completed the planned number of HBOTs from the initial diagnosis. Twenty-nine patients (11.74%) terminated the HBOT due to side effects during the treatment. The most common side effect from the HBOT was otalgia (82, 33.20%). Other side effects were chest discomfort (5, 2.02%), headache (4, 1.62%), dyspnea (3, 1.21%), anxiety (3, 1.21%), and limb pain (2, 0.81%). One hundred sixty-two patients (65.59%) completed the HBOT at initial treatment without pause, thirty-five patients (14.17%) had a pause during treatment, and fifty patients (20.24%) terminated the treatment (Table 1).

### 3.2. Events of Pause Termination According to HBOT Pressure Grade

Among the initial trials, the most frequently paused pressure occurred in the 1.2 to 1.4 grade (48, 26.67%), and the median time for patients to pause compression during treatment was 3 min after the starting treatment [2–5]. The next most common stoppage occurred in the 1.4 to 1.6 grade (38, 21.11%) and the 1.8 to 2.0 grade (29, 16.11%) in that order. In addition, among the initial trials, the most frequently terminated pressure occurred above the 2.0 grade (15, 30.00%), and the median time for all patients to discontinue treatment was 1 [1–1] minute after starting treatment. The next most terminations occurred in the 1.4 to 1.6 grade (10, 20.00%) and the 1.8 to 2.0 grade (10, 20.00%) in that order (Table 2).

### 3.3. Demographic and Clinical Differences Between Groups by HBOT Termination Pressure Grade

Demographic and clinical differences were analyzed for the group that completed treatment without intermediate termination and the group that was classified into three groups based on the pressure grade where treatment was terminated before completion (Table 3). The overall *p*-value for the proportion of patients with SSNHL between groups was statistically significant (*p* = 0.047). However, none of the groups were statistically significant in the post hoc analysis. Additionally, there was a statistically significant difference between groups in patients who complained of otalgia, one of the side effects (*p* < 0.001). In post hoc analysis, all were statistically significant for otalgia except for the 1.0~1.2 interval with *p*-values <0.001/0.7015/<0.001/<0.001 for no termination/1.0~1.2/1.2~2.0/2.0~ in that order.

### 3.4. Relationship Between the Frequency of Pauses by Pressure Grade and Side Effects

Otalgia was the most common side effect across various pressure grades: 1.0 to 1.2 (16, 19.51%), 1.2 to 1.4 (46, 56.10%), 1.4 to 1.6 (37, 45.12%), 1.6 to 1.8 (23, 28.05%), 1.8 to 2.0 (29, 35.37%), and above 2.0 (22, 26.83%). It was also statistically significant in all pressure grades. Other than otalgia, the side effects, if any, did not cause a pause. In addition, side effects other than otalgia were not statistically significant across all pressure grades (Table 4).

### 3.5. Demographic and Clinical Differences in the Otalgia Group

Significant differences were found in the proportion of compromised wound patients between groups that completed treatment and groups divided into three pressure grades of termination before completion in the overall *p*-value (*p* = 0.013). However, when post hoc analysis was performed, the compromised wound was only statistically significant in the 1.6 ~ 1.8 pressure grade, with a *p*-value of 0.02. Among patients who complained of otalgia, the overall *p*-value was significant at 0.006, but post hoc analysis showed that it was not statistically significant in all pressure grades with a history of brain disease (Table 5).

## 4. Discussion

This study aimed to analyze the characteristics and factors related to the side effects of HBOT in a computer-controlled pressurized monoplace chamber. HBOT patients were asked to stay in the monoplace chamber for 90 min, and their compliance with treatment completion was examined. We discuss the implications of these findings, focusing on the most common side effects, their incidence, and the potential factors contributing to these outcomes.

Table 1 presents the general characteristics of patients treated with the monoplace chamber for non-emergent indications. In this study, the most frequent indicator in patients who received HBOT was sudden sensorineural hearing loss (SSNHL). In 2012, the American Academy of Otolaryngology-Head and Neck Surgery (AAO-HNS) published treatment guidelines for SSNHL [18], revised in 2019. In these revised guidelines, HBOT was subdivided into ‘optional’ levels that can be combined with steroids as initial and salvage therapy [19]. Most of the patients had hypertension and diabetes mellitus in their past medical history. In addition, the most common complication from the HBOT was otalgia. This high incidence aligns with previous studies that reported that otalgia was a common side effect due to pressure changes in hyperbaric chambers [11]. Although there were no side effects, such as facial pain or seizures, in this study, there were differences between this study and other previous studies. Skevas et al. reported that negative pressure gradients caused inflammation on the mucosal surface of the paranasal sinuses and bone cavities, compressing the paranasal space and causing congestion and edema, which was accompanied by facial pain, which was relieved when the pressure disappeared [20]. Seizure rarely occurs at typical clinical treatment pressures (2 ATA to 3 ATA) and is difficult to predict individually. Heyboer 3rd et al. reported that seizure incidence is approximately 1 in about 2000 treatments [21], and Hadanny et al. also reported a low incidence of seizures in multiplace chambers [15].

Table 2 indicates the distribution of treatment pauses and terminations across different pressure grades, which provides valuable information. Most pauses occurred in the 1.2 to 1.4 ATA range, with a median pause time of 3 min. Terminations were more frequent at pressures above 2.0 ATA. Gill and Bell noted that not all patients respond similarly to the same pressure, so an individualized treatment approach is more important than adjusting protocols based on generalized data [22]. Understanding these pressure-related trends can guide modifications in HBOT protocols to minimize side effects and improve patient outcomes.

Table 3 highlights demographic and clinical differences between groups based on the pressure at which treatment was paused or terminated. For instance, SSNHL patients showed a statistically significant difference in treatment outcomes, particularly at higher pressures (above the 2.0 grade). Hadanny et al. reported that it was particularly critical given that the median pause time in the 1.2 to 1.4 ATA range was manageable. However, higher pressures above 2.0 ATA correlate with more frequent treatment terminations due to side effects [23]. Additionally, Rozbicki et al. emphasized the importance of timely initiation and careful pressure management in improving hearing outcomes in patients with SSNHL, noting that higher pressures are often associated with increased side effects, including barotrauma and oxygen toxicity [24]. This finding may suggest that SSNHL patients are more susceptible to pressure-related side effects, necessitating tailored protocols for this subgroup.

Moreover, patients’ compliance with HBOT is significantly influenced by the occurrence of side effects. Table 4 describes side effects according to HBOT pressure grade; otalgia was the most common side effect of treatment pauses or termination across various pressure grades. The most common reason for otalgia would be considered to be due to failure in equalizing. Hwang et al. proposed the developed algorithm, which determined and equalized the unbalanced pressure of a subject based on their tympanic admittance and was evaluated in conjunction with conventional HBOT in an experiment involving 100 subjects [25]. Even if treatment was paused at a pressure level of 2.0 or higher, the possibility of undiagnosed claustrophobia or panic attacks might be presumed to be the cause of headache complaints. Miller et al. noted that claustrophobia might be managed with coaching and anxiolytic medications, and intolerance of a monoplace chamber may warrant referral to the closest multiplace chamber facility [26]. Managing and mitigating these side effects could be essential to increasing patient compliance and completing HBOT sessions.

## 5. Conclusions

This study underscores the importance of recognizing and addressing the side effects associated with HBOT in monoplace chambers. For the 50 terminated patients, the causes of the termination are unknown due to the lack of description (38 patients), the patient’s refusal of treatment (10 patients), and a problem with the ear (2 patients). Since this study is a retrospective study, defining the definite cause of the termination was limited. For those two otalgia patients, one was diagnosed with tympanic membrane effusion; the ENT recommended the cessation of HBOT. The other patient was diagnosed with otitis media externa, and HBOT was stopped after tympanostomy tube insertion. Another limitation is that our indications of hyperbaric therapy were not evenly distributed. SSNHL patients already received intra-tympanic injections, which might also have affected patients having more trouble with equalization. Middle ear barotrauma is the most prevalent complication, highlighting the need for effective pressure equalization techniques and patient education. While oxygen toxicity is rare, monitoring remains critical. Tailoring HBOT protocols based on patient demographics and clinical characteristics, especially for conditions like SSNHL, can enhance treatment safety and efficacy. Future studies should focus on preventive strategies and protocol optimization to reduce side effects and improve patient adherence to HBOT.

## Figures and Tables

**Table 1:** General characteristics of patients treated with monoplace chamber for non-emergent indications.

Variables	Total Patients (*n* = 247)
Age, yr (M ± S.D)	59.35 ± 15.05
Male, *n* (%)	157 (63.56)
Indication	
DNS from ACOP	5 (2.02)
Gas gangrene	2 (0.81)
DM foot	24 (9.72)
Ischemic limb (Buerger’s Dz and PAOD)	11 (4.45)
SSNHL	69 (27.94)
Radiation injury	31 (12.55)
Burn	4 (1.62)
Graft/Flap	60 (24.29)
Compromised wound (degloving, acute wound)	30 (12.15)
Chronic osteomyelitis	10 (4.05)
Other (non-indication spinal cord injury)	1 (0.40)
Past Medical Hx., *n* (%)	
None	47 (19.03)
HTN	114 (46.15)
DM	97 (39.27)
Ear disease	3 (1.21)
Respiratory system (asthma, COPD, pneumothorax)	8 (3.24)
Cardiovascular system (coronary disease, heart failure)	18 (7.29)
Genitourinary system	26 (10.53)
Eye disease	6 (2.43)
Nervous system	15 (6.07)
Mental and behavioral disorders (claustrophobia, panic disorder, anxiety disorder)	5 (2.02)
Malignancy	38 (15.38)
Others	83 (33.20)
Mental Status, *n* (%)	
Alert	242 (97.98)
Verbal	5 (2.02)
Final outcome of completed HBOT session, *n* (%)	
Number of Total HBOT completed	137 (55.47)
Therapy terminated due to complications	29 (11.74)
Follow up loss	29 (11.74)
Terminated by physician	8 (3.24)
Transfer out	13 (5.26)
Patient refused due to personal reason	31 (12.55)
Side Effect	
Otalgia	82 (33.20)
Headache	4 (1.62)
Dyspnea	3 (1.21)
Chest discomfort	5 (2.02)
Anxiety	3 (1.21)
Limb pain	2 (0.81)
HBOT outcome at initial treatment	
Completed without pause	162 (65.59)
Completed with pause	35 (14.17)
Treatment terminated	50 (20.24)

M—mean, S.D—standard deviation, DNS—delayed neuropsychiatric sequelae, ACOP—acute carbon monoxide poisoning, DM—diabetes mellitus, Dz—disease, PAOD—peripheral arterial disease, SSNHL—sudden sensorineural hearing loss, HBOT—hyperbaric oxygen therapy, Hx—history, HTN—hypertension, COPD—chronic obstructive pulmonary disease.

**Table 2:** Distribution of the delayed treatment time by paused or terminated pressure.

Event	Pressure (ATA)	Frequency (*n*, %)	Delayed Treatment Time (min)		
			Minimum	Maximum	Median [IQR]
HBOT paused (*n* = 180)	1.0~1.2	17 (9.44)	1	8	2 [1–3]
	1.2~1.4	48 (26.67)	1	15	3 [2–5]
	1.4~1.6	38 (21.11)	1	13	2 [1–4]
	1.6~1.8	23 (12.78)	1	14	1 [1–2]
	1.8~2.0	29 (16.11)	1	7	2 [1–2]
	2.0~	25 (13.89)	1	10	3 [2–5]
HBOT terminated (*n* = 50)	1.0~1.2	1 (2.00)	1	1	1 [1–1]
	1.2~1.4	9 (18.00)	1	9	2 [1–4]
	1.4~1.6	10 (20.00)	1	4	1 [1–1]
	1.6~1.8	5 (10.00)	1	1	2 [1–2]
	1.8~2.0	10 (20.00)	1	4	3 [2–7]
	2.0~	15 (30.00)	1	9	1 [1–1]

Available for several responses in each pressure section. ATA—atmospheric absolute pressure, IQR—interquartile range.

**Table 3:** Clinical difference between groups by HBOT termination pressure grade.

Variables	Pressure Grade				*p*-Value
	No Terminated (*n* = 197)	1.0~1.2 (*n* = 1)	1.2~2.0 (*n* = 34)	2.0~ (*n* = 15)	
Age, yr [IQR]	60 [47–70]	64 [64–64]	61.5 [54–72]	61 [53–69]	0.868
Male, *n* (%)	129 (65.48)	0	20 (58.82)	8 (53.33)	0.345
Indication, *n* (%)					
DNS from ACOP	4 (2.03)	0	1 (2.94)	0	0.681
Gas gangrene	2 (1.02)	0	0	0	1.000
DM foot	21 (10.66)	0	3 (8.82)	0	0.629
Ischemic limb	10 (5.08)	0	1 (2.94)	0	1.000
SSNHL	51 (25.89)	1 (100.00)	9 (26.47)	8 (53.33)	0.047 *
Radiation injury	27 (13.71)	0	3 (8.82)	1 (6.67)	0.738
Burn	3 (1.52)	0	0	1 (6.67)	0.316
Graft/Flap	47 (22.22)	0	9 (26.47)	4 (26.67)	0.911
Compromised wound	24 (12.18)	0	6 (17.65)	0	0.337
Chronic osteomyelitis	8 (4.06)	0	1 (2.94)	1 (6.67)	0.679
Other	0	0	1 (2.94)	0	0.202
Past Medical Hx., *n* (%)					
None	38 (19.29)	0	5 (14.71)	4 (26.67)	0.680
HTN	91 (46.19)	1 (100.00)	18 (52.94)	4 (26.67)	0.462
DM	83 (42.13)	0	11 (32.35)	3 (20.00)	0.203
Ear disease	2 (1.02)	0	1 (2.94)	0	0.494
Respiratory system	6 (3.05)	0	2 (5.88)	0	0.613
Cardiovascular system	14 (7.11)	0	3 (8.82)	1 (6.67)	0.900
Genitourinary system	22 (11.17)	0	3 (8.82)	1 (6.67)	1.000
Eye disease	6 (3.05)	0	0	0	0.731
Brain	10 (5.08)	0	5 (14.71)	0	0.161
Mental and behavioral disorders	4 (2.03)	0	1 (2.94)	0	0.681
Malignancy	34 (17.26)	0	3 (8.82)	1 (6.67)	0.476
Others	62 (31.47)	0	14 (41.18)	6 (40.00)	0.568
Complication, *n* (%)					
Otalgia	32 (16.24)	1 (100.00)	33 (97.06)	15 (100.0)	<0.001 ***
Headache	3 (1.52)	0	1 (2.94)	0	0.598
Dyspnea	3 (1.52)	0	0	0	1.000
Chest discomfort	3 (1.52)	0	0	0	1.000
Anxiety	3 (1.52)	0	0	0	1.000
Limb pain	2 (1.02)	0	0	0	1.000

* *p* < 0.05, ** *p* < 0.01, *** *p* < 0.001. IQR—interquartile range, DNS—delayed neuropsychiatric sequelae, ACOP—acute carbon monoxide poisoning, DM—diabetes mellitus, SSNHL—sudden sensorineural hearing loss, Hx—history, HTN—hypertension.

**Table 4:** Relationship between the frequency of pauses by pressure grade and side effects.

Patients No. of Side Effects	Events of HBOT Pause by Pressure Grade (ATA)						
		1.0~1.2 (*n* = 17)	1.2~1.4 (*n* = 48)	1.4~1.6 (*n* = 38)	1.6~1.8 (*n* = 24)	1.8~2.0 (*n* = 29)	2.0~ (*n* = 25)
Otalgia (*n* = 82)	*n*, %	16 (19.51)	46 (56.10)	37 (45.12)	23 (28.05)	29 (35.37)	22 (26.83)
	*p*-value	<0.001 ***	<0.001 ***	<0.001 ***	<0.001 ***	<0.001 ***	<0.001 ***
Headache (*n* = 4)	*n*, %	0	1 (25.00)	1 (25.00)	0	0	2 (50.00)
	*p*-value	1.000	0.581	0.490	1.000	1.000	0.052
Dyspnea (*n* = 3)	*n*, %	0	1 (33.33)	0	0	0	0
	*p*-value	1.000	0.479	1.000	1.000	1.000	1.000
Chest discomfort (*n* = 5)	*n*, %	0	1 (20.00)	0	1 (20.00)	0	2 (40.00)
	*p*-value	1.000	1.000	1.000	0.389	1.000	0.081
Anxiety (*n* = 3)	*n*, %	0	0	0	0	0	1 (33.33)
	*p*-value	1.000	1.000	1.000	1.000	1.000	0.275
Limb pain (*n* = 2)	*n*, %	1 (50.00)	0	0	0	0	0
	*p*-value	0.133	1.000	1.000	1.000	1.000	1.000

* *p* < 0.05, ** *p* < 0.01, *** *p* < 0.001.

**Table 5:** Demographic and clinical differences in the otalgia group by HBOT termination pressure grade in the otalgia group.

Variables	Pressure Grade							*p*-Value
	No Terminated (*n* = 32)	1.0~1.2 (*n* = 1)	1.2~1.4 (*n* = 9)	1.4~1.6 (*n* = 10)	1.6~1.8 (*n* = 5)	1.8~2.0 (*n* = 10)	2.0~ (*n* = 15)	
Age, yr [IQR]	59.5 [56.5–71.5]	64 [64–64]	57 [48–76]	59 [53–62]	63 [62–68]	64 [56–73]	61 [53–69]	0.861
Male, *n* (%)	17 (53.13)	0	5(55.56)	7 (70.00)	3 (60.00)	5 (50.00)	8 (53.33)	0.943
Indication, *n* (%)								
DNS from ACOP	3 (9.38)	0	0	0	0	1 (10)	0	0.804
Gas gangrene	0	0	0				0	-
DM foot	6 (18.75)	0	0	0	1 (20.00)	2 (20)	0	0.219
Ischemic limb	1 (3.13)	0	0	1	0	0	0	0.706
SSNHL	7 (21.88)	1 (100.00)	2 (22.22)	3 (30.00)	1 (20.00)	3 (30)	8 (53.33)	0.288
Radiation injury	3 (9.38)	0	0	2 (20.00)	0	1 (10)	1 (15)	0.853
Burn	0	0	0	0	0	0	1 (6.67)	0.610
Graft/Flap	10 (31.25)	0	5 (55.56)	2 (20.00)	0	2 (20)	4 (26.67)	0.445
Compromised wound	2 (6.25)	0	1	2 (20.00)	3 (60.00)	0	0	0.013 *
Chronic osteomyelitis	0	0	1 (11.11)	0	0	0	1 (6.67)	0.288
Other	0	0	0	0	0	1 (10)	0	0.427
Past Medical Hx., *n* (%)								
None	3 (9.38)	0	1 (11.11)	2 (20.00)	1 (20.00)	1 (10)	4 (26.67)	0.684
HTN	19 (59.38)	1 (100.00)	6 (66.67)	4 (40.00)	3 (60.00)	5 (50)	4 (26.67)	0.313
DM	19 (59.38)	0	2 (22.22)	2 (20.00)	3 (60.00)	4 (40)	3 (20)	0.055
Ear disease	0	0	0	0	0	1 (10)	0	0.427
Respiratory system	0	0	1 (11.11)	1 (10.00)	0	0	0	0.186
Cardiovascular system	5 (15.63)	0	0	1 (10.00)	2 (40.00)	0	1 (6.67)	0.307
Genitourinary system	7 (21.88)	0	0	1 (10.00)	2 (40.00)	0	1 (6.67)	0.213
Eye disease	2 (6.25)	0	0	0	0	0	0	1.000
Brain	0	0	1 (11.11)	2 (20.00)	2 (40.00)	0	0	0.006 **
Mental and behavioral disorders	0	0	0	0	0	1 (10)	0	0.427
Malignancy	5 (15.63)	0	0	2 (20.00)	0	1 (10)	1 (6.67)	0.822
Others	13 (40.63)	0	3 (33.33)	5 (50.00)	2 (40.00)	4 (40)	6 (40)	0.995

* *p* < 0.05, ** *p* < 0.01, *** *p* < 0.001, IQR—interquartile range, DNS—delayed neuropsychiatric sequelae, ACOP—acute carbon monoxide poisoning, DM—diabetes mellitus, SSNHL—sudden sensorineural hearing loss, Hx—history, HTN—hypertension.

## Data Availability

The data that support the findings of this study are available on request from the corresponding author. The data is not publicly available due to privacy or ethical restrictions.
